# Supplementary material for: Architecture of the Saccharomyces cerevisiae NuA4/TIP60 complex
Source: Nat Commun. 2018 Mar 20;9:1147. doi: 10.1038/s41467-018-03504-5 (PMC5861120; doi:10.1038/s41467-018-03504-5)
Supplement: Supplementary file 1 — Mec1/ATR (PDB ID 5X6O) 8 are color-coded [file 41467_2018_3504_MOESM1_ESM.pdf]

Architecture of the *Saccharomyces cerevisiae* NuA4/TIP60 complex

Wang *et al.*

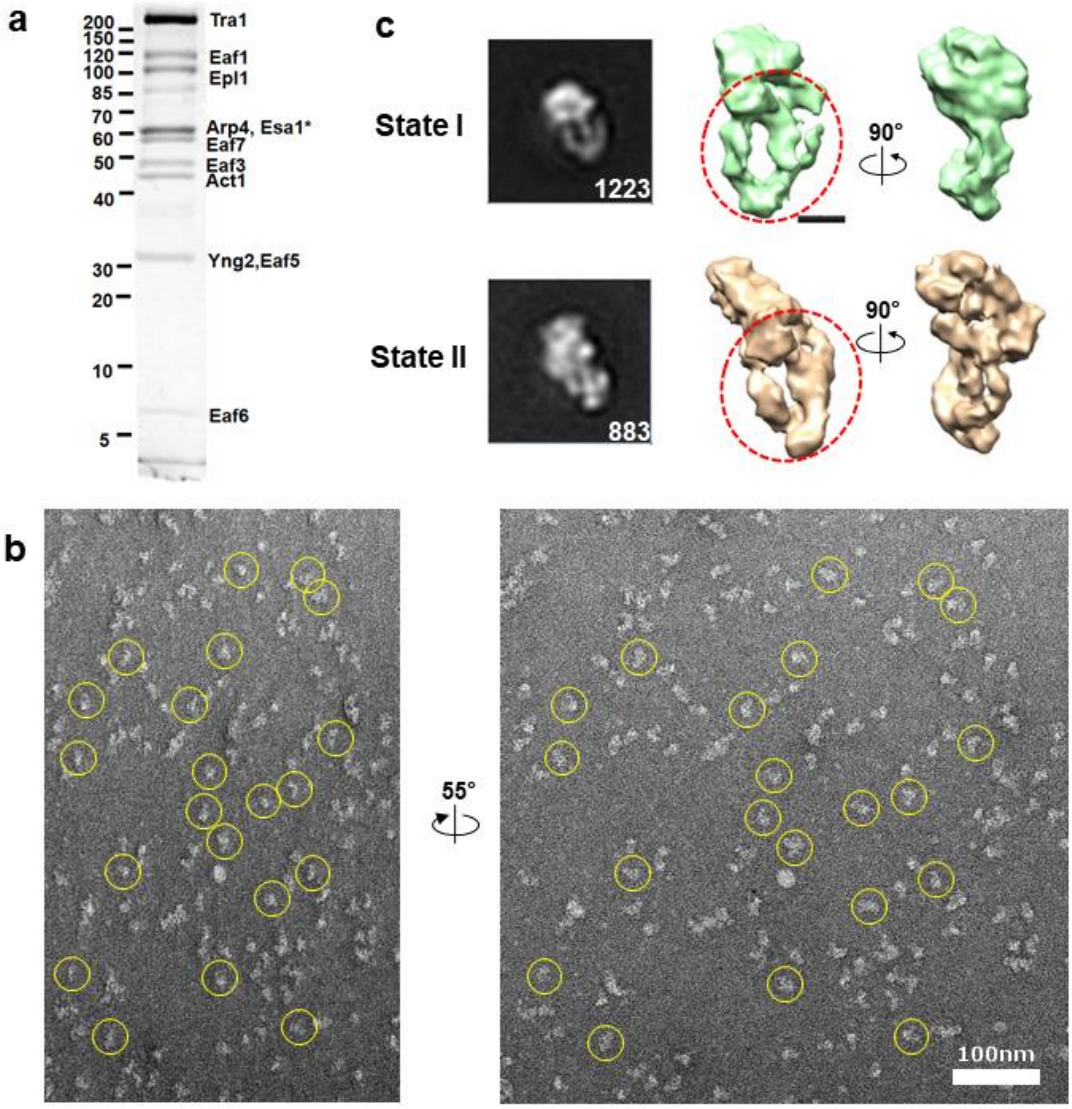

Supplementary Figure 1 | Preparation and EM analysis of the endogenous NuA4 complex. a, SDS-PAGE analysis of the NuA4 complex endogenously purified from the *S. cerevisiae*. **b**, Tilt pair (0° and -55°) micrographs of a negatively stained specimen. Raw images are overlaid with circles marking the corresponding particles picked from the pair of micrographs. Scale bar, 100 nm. **c**, 2D and 3D structures of the two states of NuA4 calculated by the random conical tilt (RCT) method [1]. The red ellipses denote the putative Tra1 density. All the 3D structures are prepared by Chimera [2]. Scale bar, 50Å.

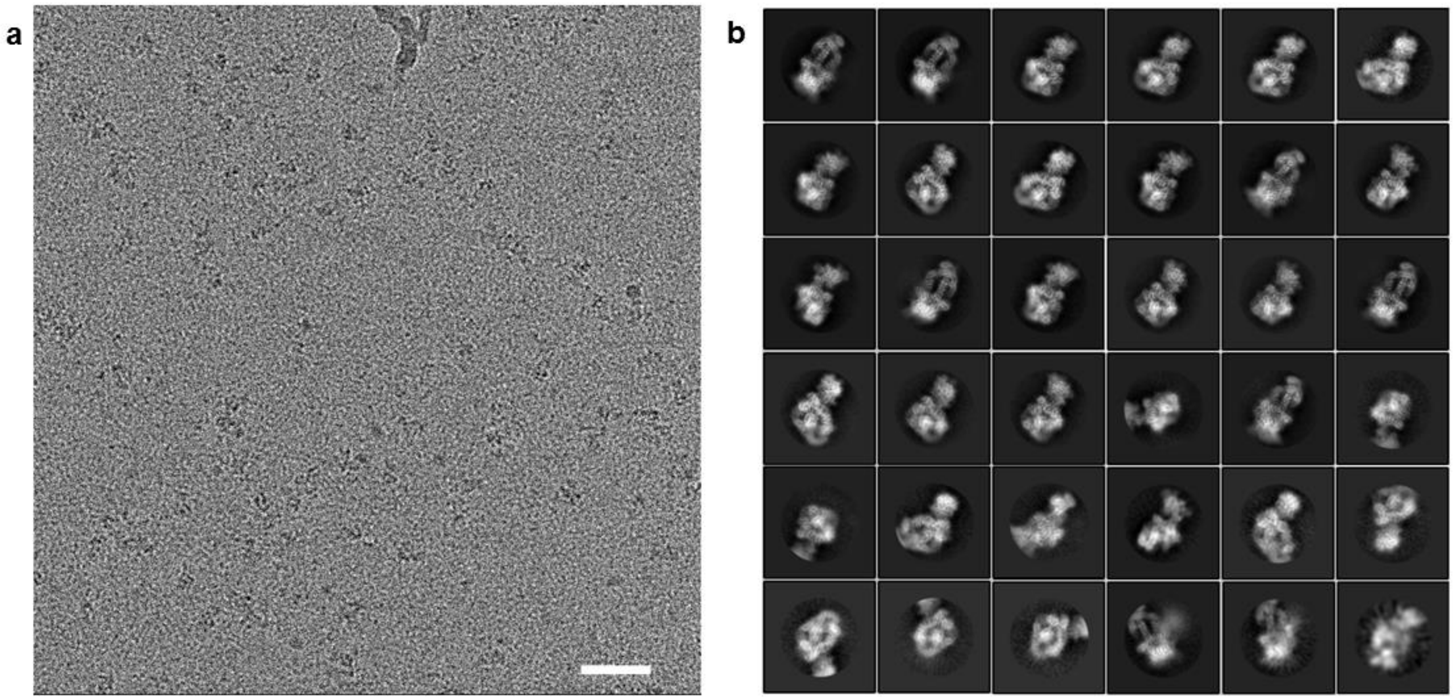

Supplementary Figure 2 | Cryo-EM analysis of the NuA4 complex. a, A typical micrograph of the NuA4 complex preserved in vitrified ice. Scale bar, 10 nm. **b**, Typical 2D class averages obtained after reference-free alignment and classification of images of NuA4 particles.

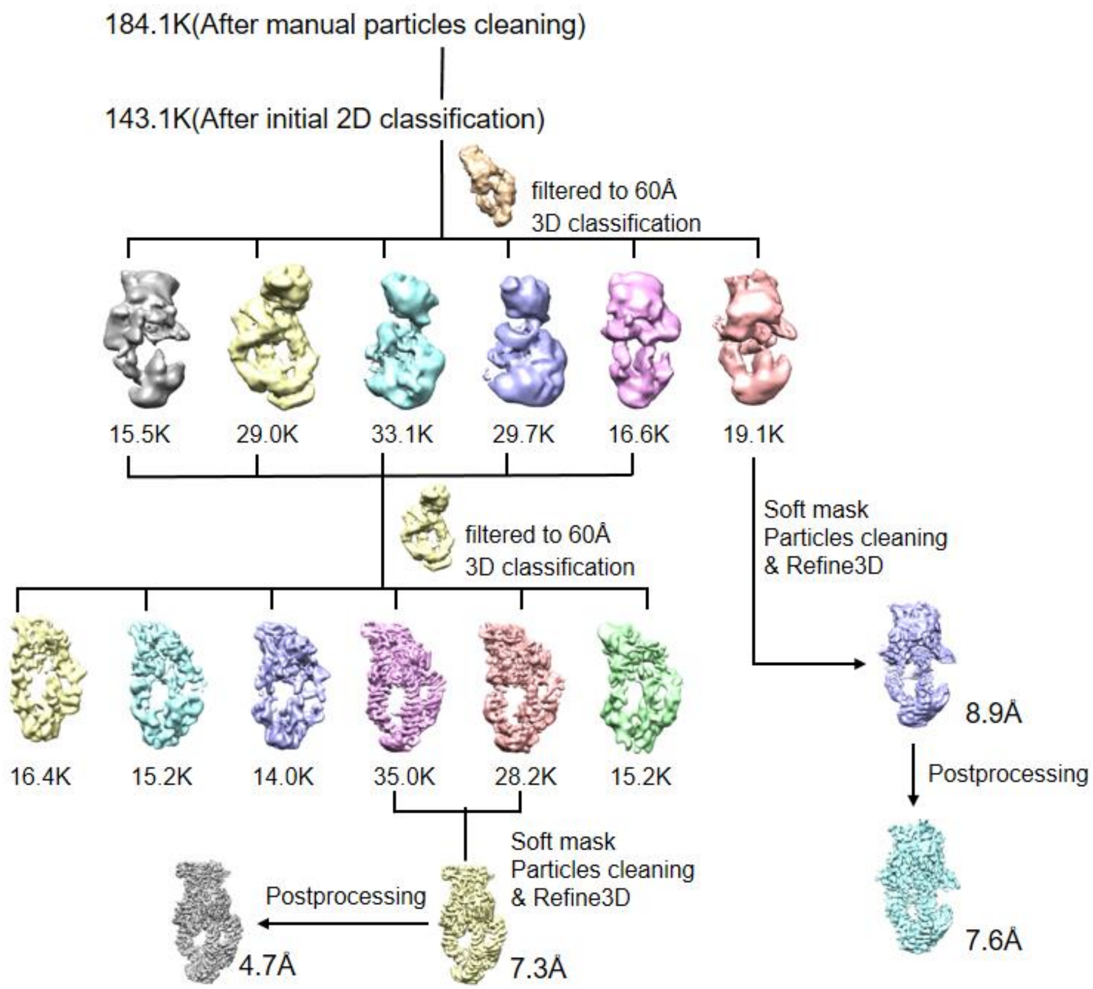

Supplementary Figure 3 | 3D reconstruction of the NuA4 complex. The schematic diagram of cryo-EM data processing of the NuA4, including particles selection, classification and 3D refinement. Details are provided in the Method section.

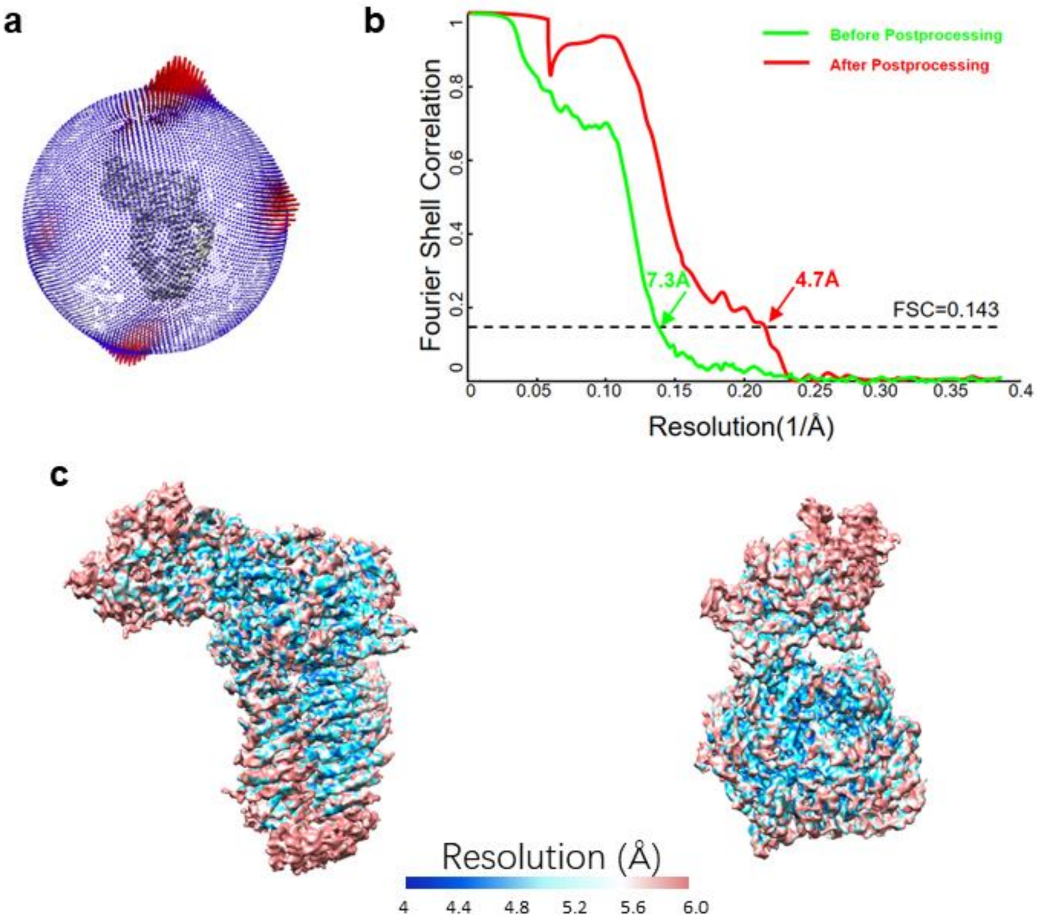

Supplementary Figure 4 | Resolution and validation of the cryo-EM map of the TEEAA assembly. a, Angular distribution for the final reconstruction of the NuA4 TEEAA sub-complex. Each cylinder represents one view and the height of the cylinder is proportional to the number of particles for that view. **b,** FSC curve for the cryo-EM density map according to the gold-standard criterion [3]. The final resolution is 4.7 Å. **c,** Front and top views of the 3D density map colored according to local resolution estimated by ResMap [4].

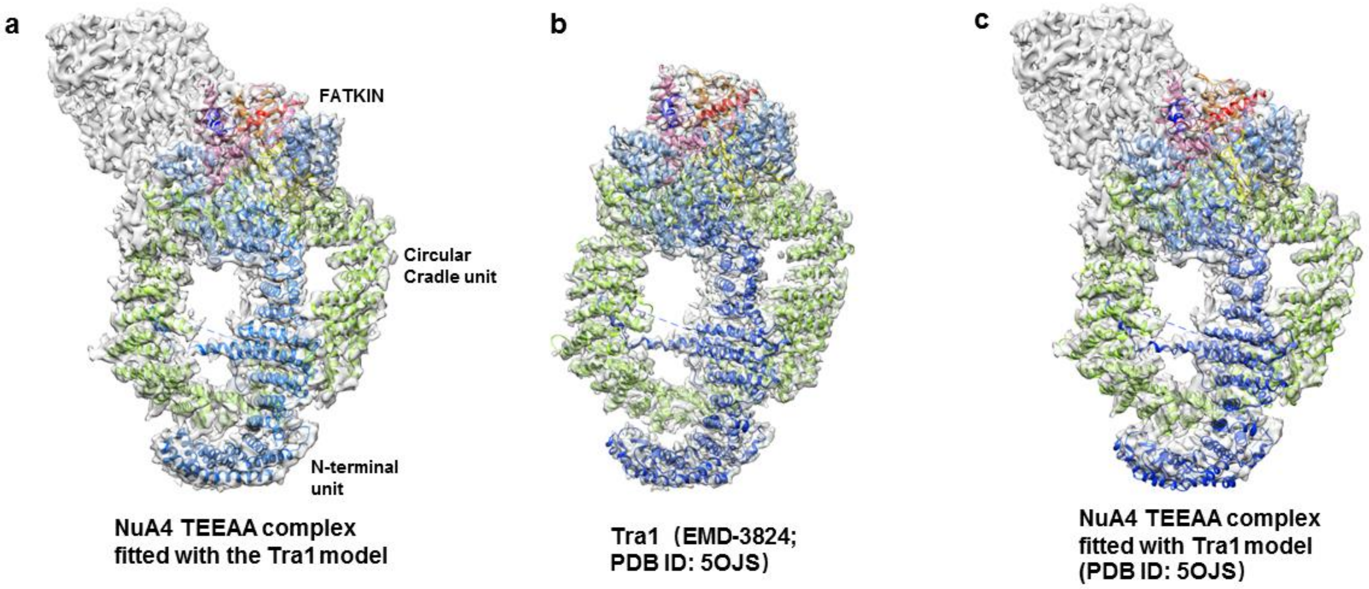

Supplementary Figure 5 | Structural comparison of Tra1 assembled into the NuA4 complex and the *apo* state. a, Cryo-EM structure of NuA4 TEEAA sub-complex fitted with our Tra1 model. **b,** CryoEM structure of Tra1 (EMD-3824) fitted with its model (PDB ID :5OJS) [5]. **c,** CryoEM structure of NuA4 TEEAA sub-complex fitted with the published model (PDB ID :5OJS). The correlation coefficient of fitting Tra1 (EMD 3824, PDB ID: 5OJS) into our Cryo-EM map is 0.85. The color scheme of Tra1 is the same as in Fig. 3b.

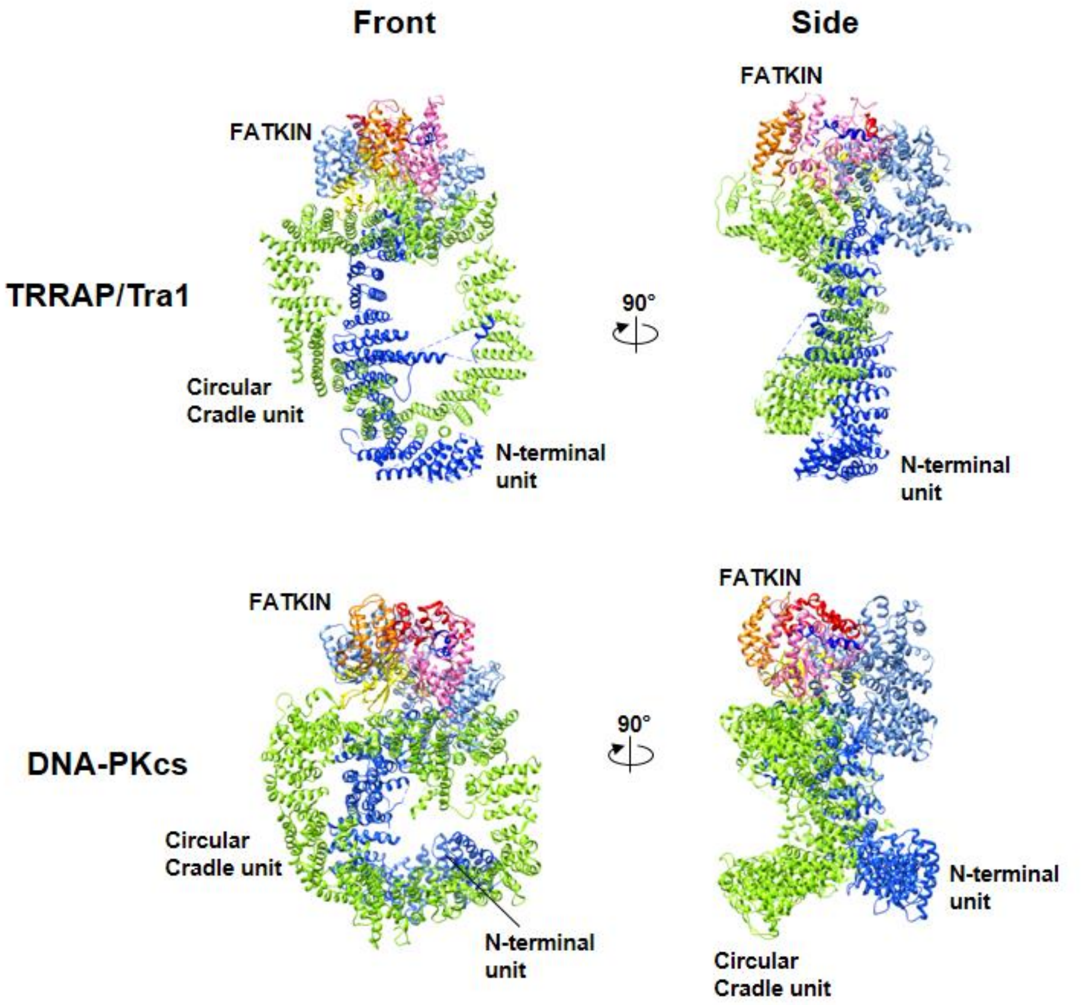

Supplementary Figure 6 | Comparison of the architecture of the Tra1 with that of the DNA-PKcs. Front and side views of Tra1 and DNA-PKcs [6] are shown, with domain organization and color assignment as Fig. 3b.

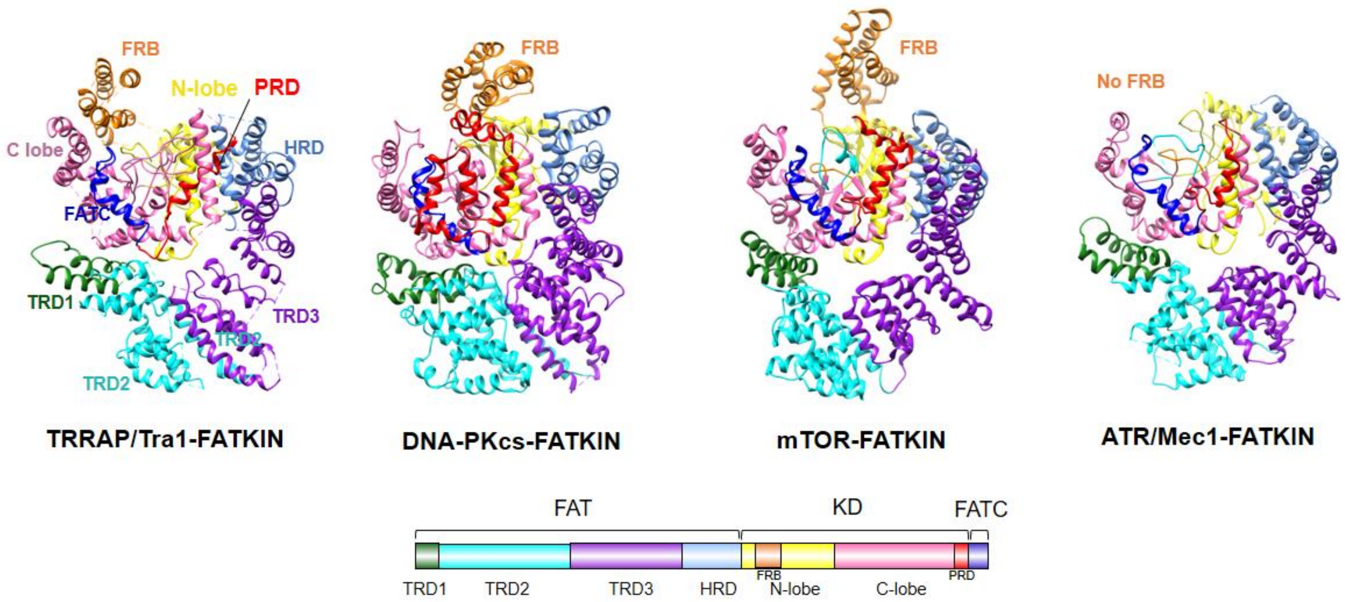

Supplementary Figure 7 | Structural comparison of the FATKIN domain of the Tra1 with that of the DNA-PKcs, mTOR and Mec1/ATR. The FATKIN domain of Tra1, DNA-PKcs (PDB ID 5LUQ) [6], mTOR (PDB ID 4JSV) [7] and Mec1/ATR (PDB ID 5X6O) [8] are color-coded by domain assignment. The domain organization and color scheme is shown at bottom. The position of FRB insertion in Tra1 is very different from that in DNA-PKcs and mTOR, while no FRB exists in Mec1/ATR.

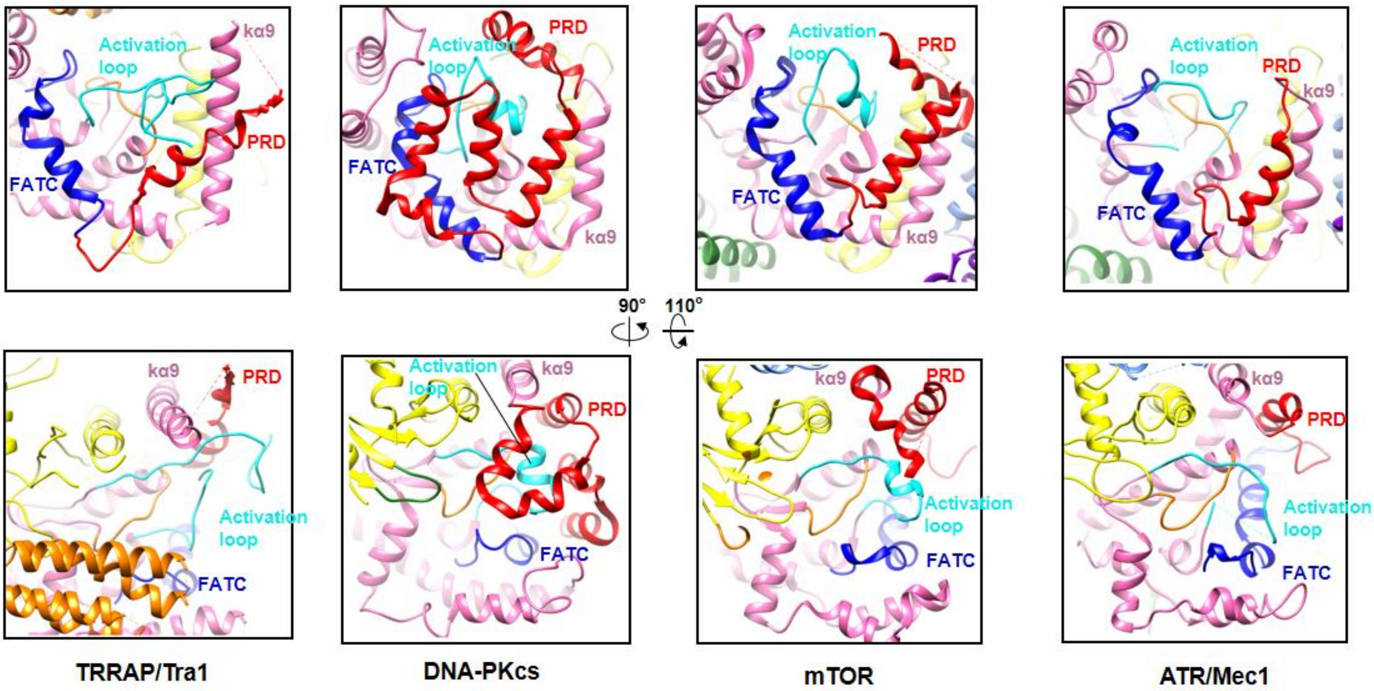

Supplementary Figure 8 | Structural comparison of the kinase active site of the Tra1 with that of the DNA-PKcs, mTOR and Mec1/ATR. Two detailed views of the active site are rotated as indicated. The kinase with the N-lobe is shown in yellow, the C-lobe is in hot pink, the FRB insertion in orange, PRD in red and FATC is in blue. The activation loop is colored in cyan, the catalytic loop in orange. The Tra1 PRD domain moves outwards by 45° relative to the kα9, whereas the PRD domain in DNA-PKcs, mTOR and Mec1/ATR is almost paralleled with kα9.

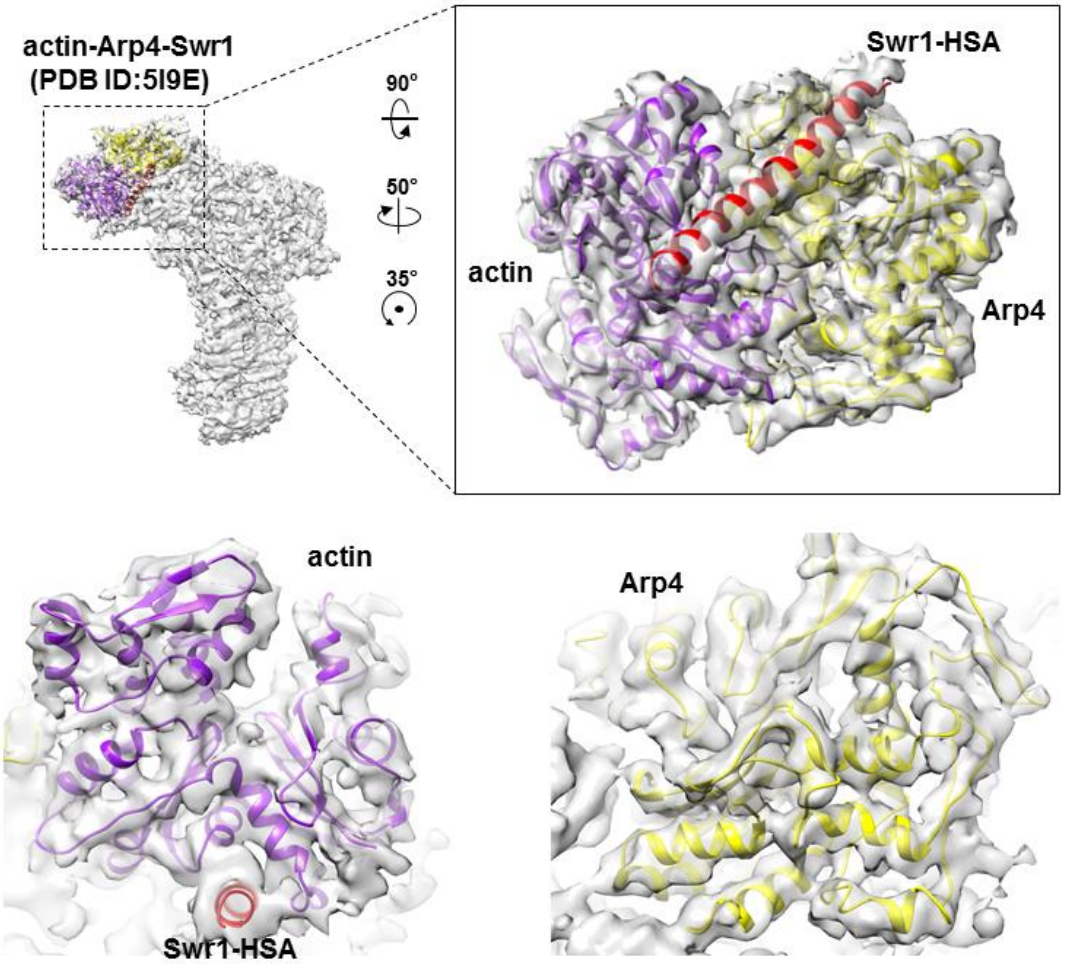

Supplementary Figure 9 | Fitting of the structure of actin-Arp4-Swr1 HSA. The crystal structure of actin-Arp4-Swr1 HSA (PDB ID:5I9E) was fitted into the cryo-EM density map. The correlation coefficient of fitting actin-Arp4-Swr1 HSA (PDBID: 5I9E) is 0.63.

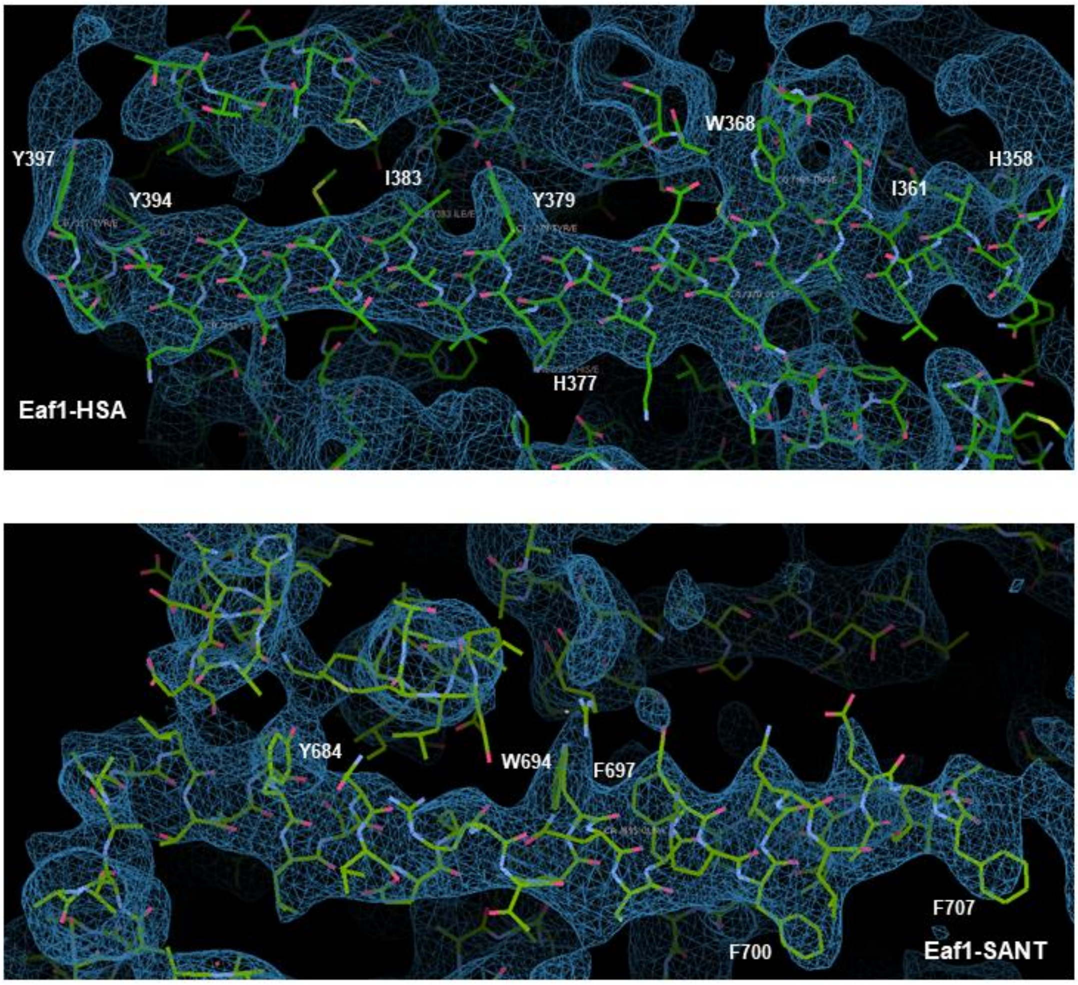

Supplementary Figure 10 | The side-chains in the cryo-EM density map in Eaf1 HSA (top) and SANT (bottom) domain.

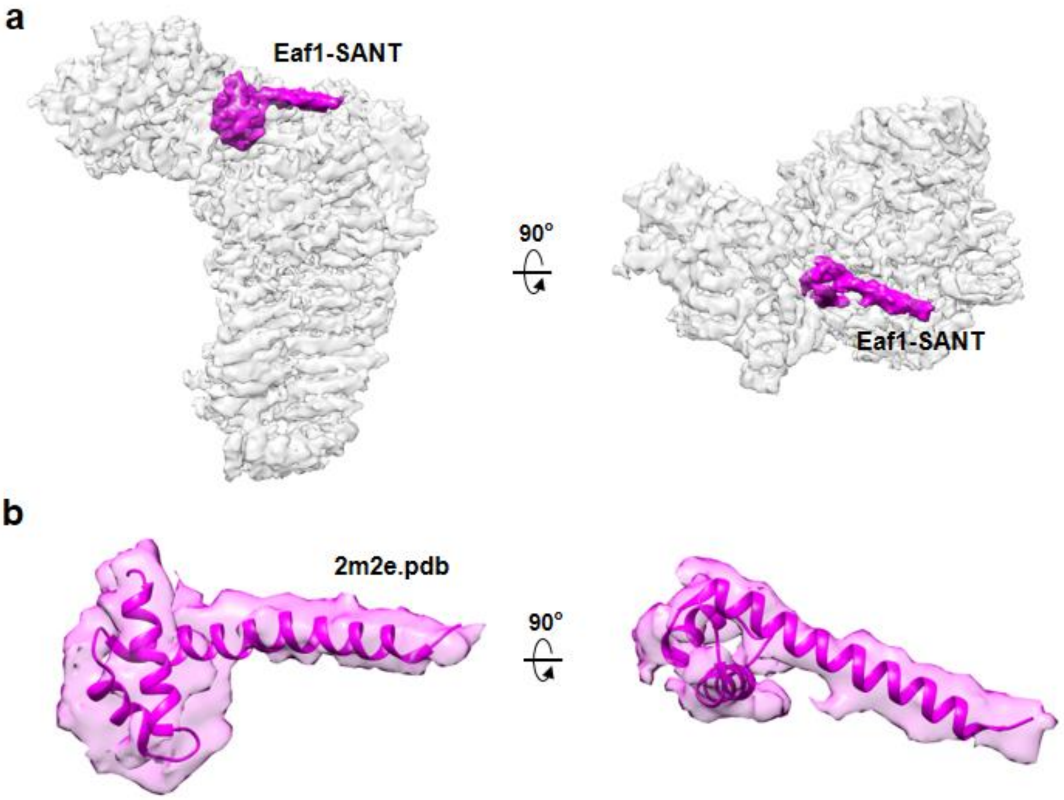

Supplementary Figure 11 | Structure of Eaf1-SANT domain. a, The TEEAA cryo-EM density is shown as a translucent surface and the SANT domain is shown as a solid magenta surface. **b,** The NMR structure of the SANT domain of human DNAJC2 (PDB ID 2M2E) was fitted into the corresponding cryo-EM density.

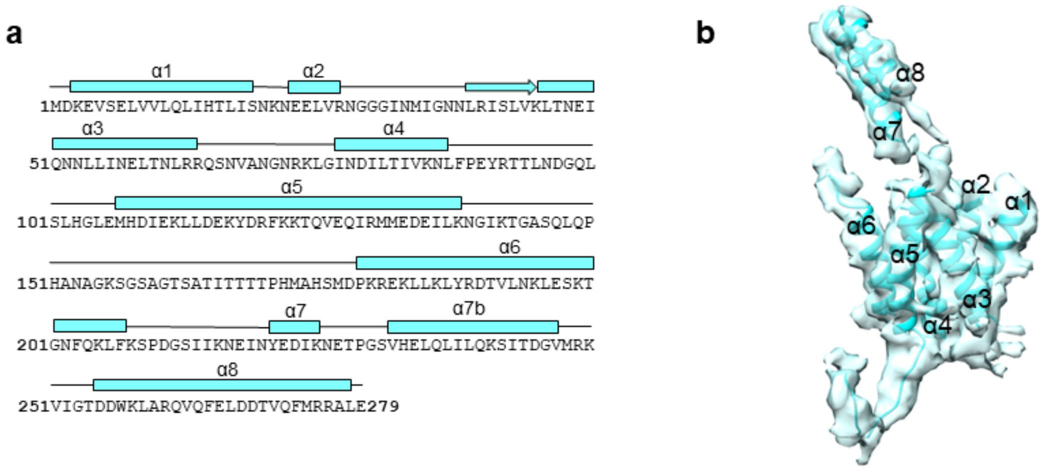

Supplementary Figure 12 | Structure of Eaf5 subunit. a, Sequence and secondary structure of Eaf5 subunit. Helices are indicated as rounded cylinders, β strands as arrows, segments lacking regular secondary structure as solid lines. **b,** The topology of the Eaf5 subunit.

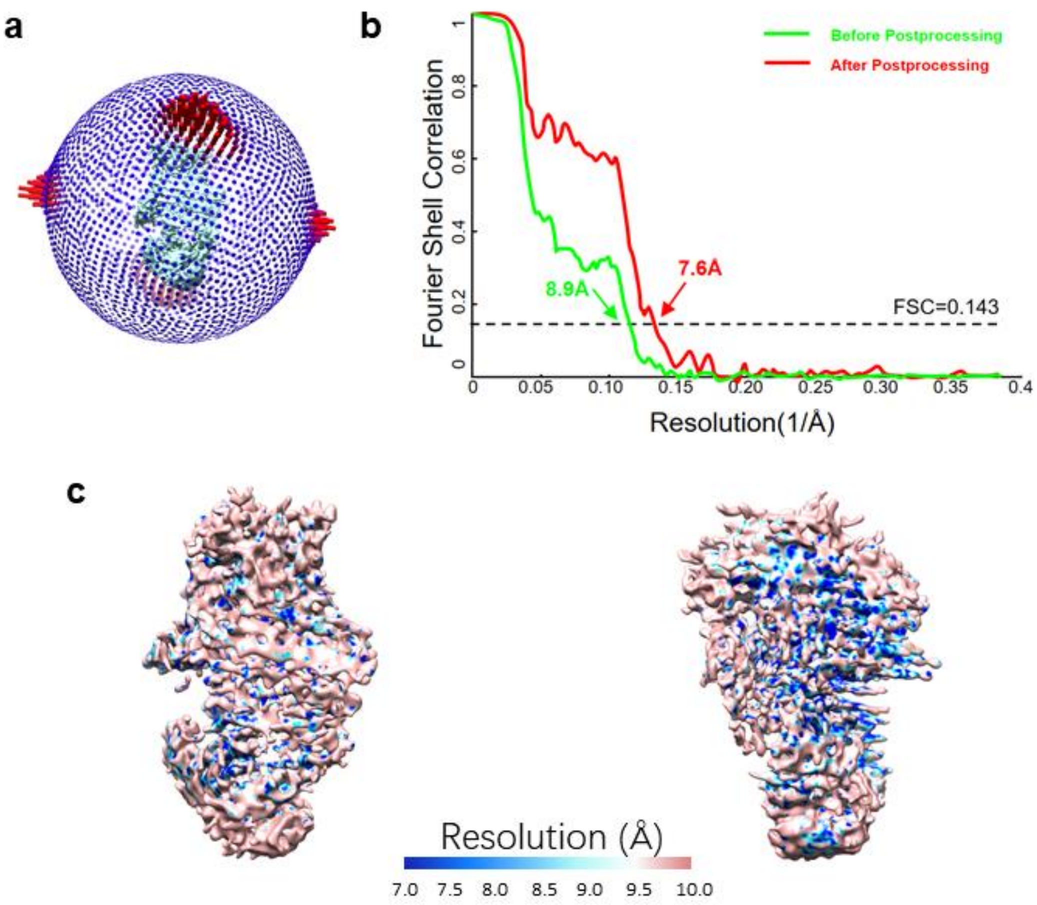

Supplementary Figure 13 | Resolution and validation of cryo-EM map of the NuA4 TEEAA-piccolo assembly. a, Angular distribution for the final reconstruction of the NuA4 TEEAA-piccolo assembly. Each cylinder represents one view and the height of the cylinder is proportional to the number of particles for that view. **b,** FSC curve for the cryo-EM density map according to the gold-standard criterion [3]. The final resolution is 7.6 Å. **c,** Front and side views of the 3D density map colored according to local resolution estimated by ResMap [4].

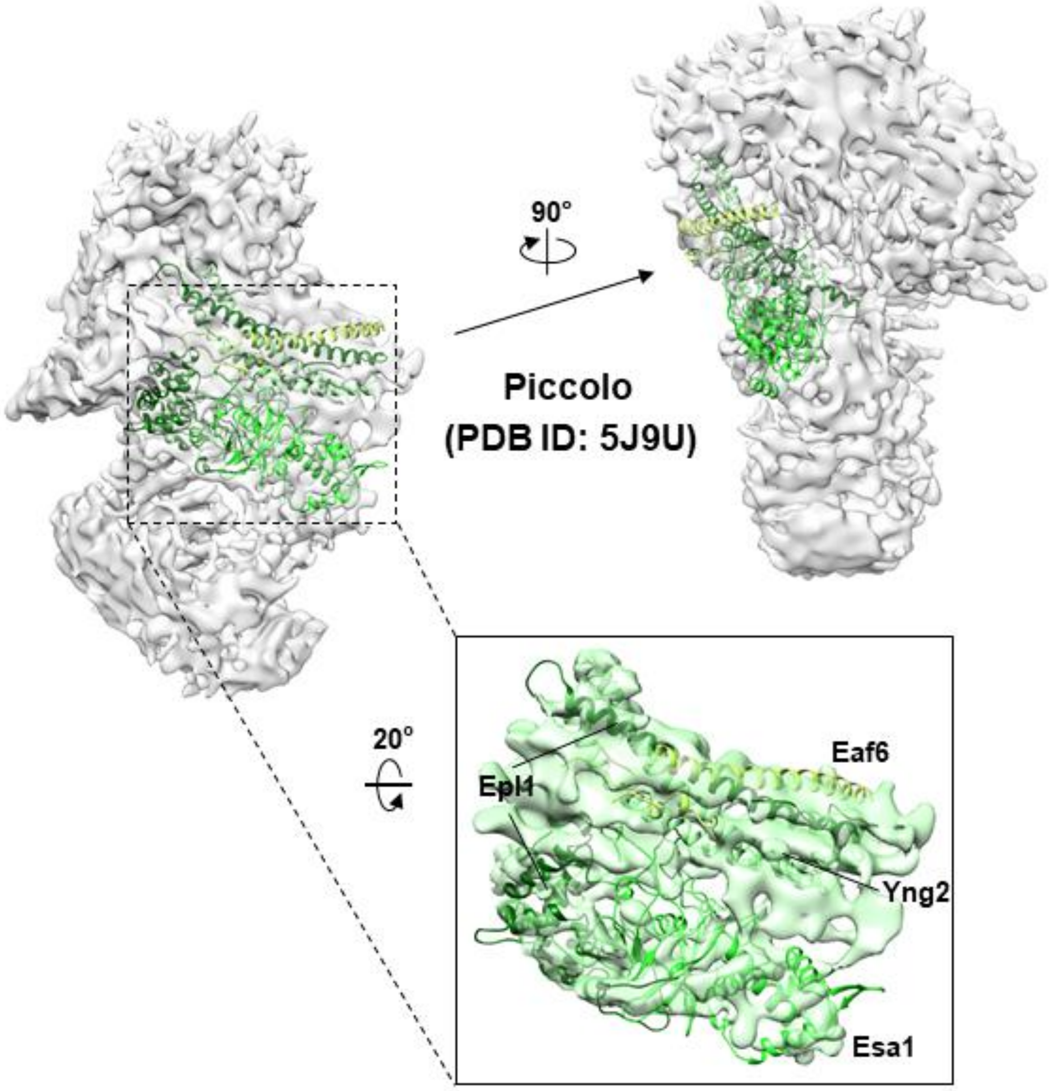

Supplementary Figure 14 | Fitting of the structure of the piccolo. The crystal structure of NuA4 piccolo (PDB ID:5J9U) was splitted into two parts at the flexible linker (Epl1 aa 320) and separately fitted into the cryo-EM map as two rigid bodies. The correlation coefficient value of fitting four helices bundle part and fitting core part (Esa1 and Eaf1) into our cryo-EM map is 0.85 and 0.72, respectively.

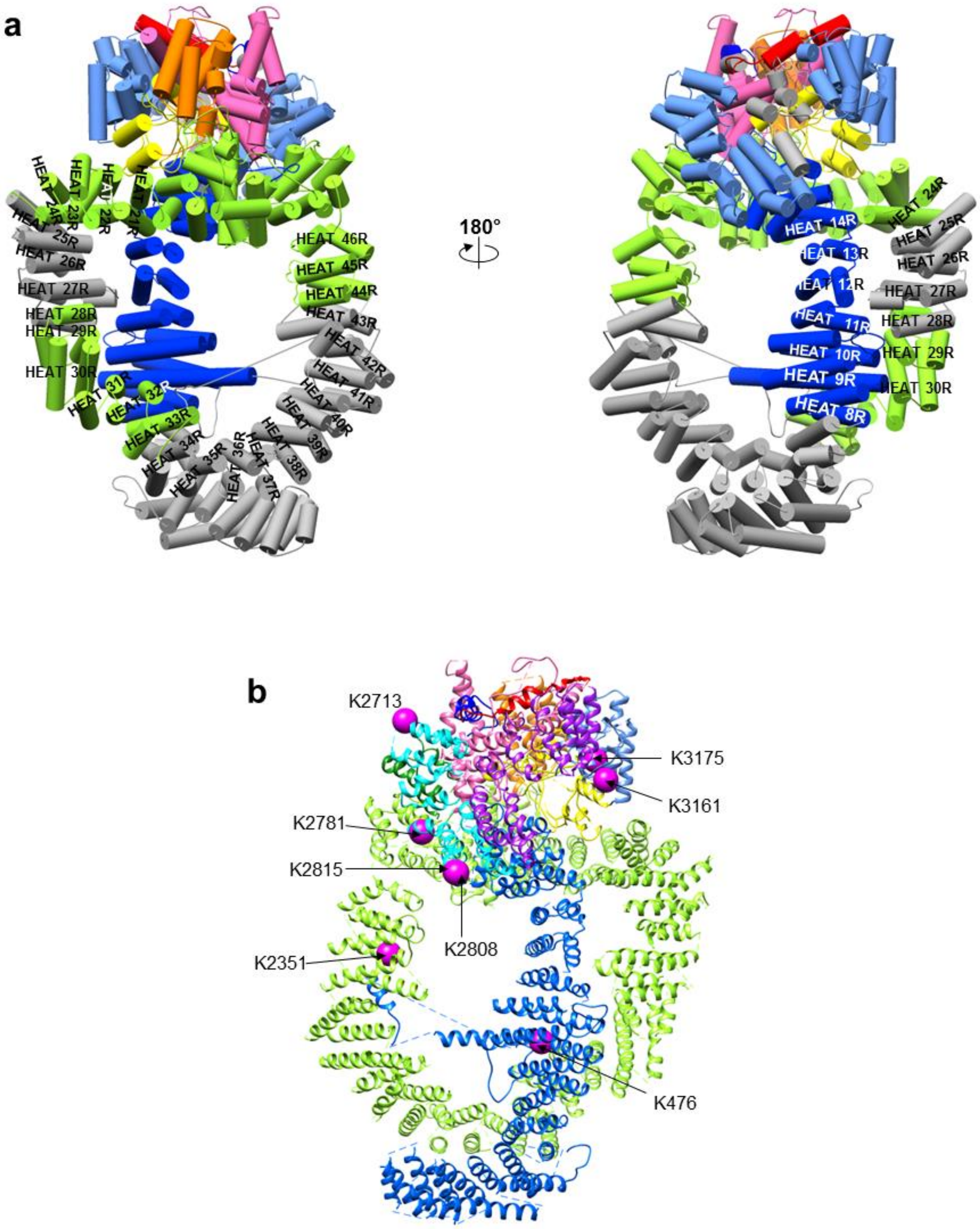

Supplementary Figure 15 | Scaffolding function of the Tra1 subunit. a, Mapping critical Tra1 deletions [9] on the Tra1 structural model with the HEAT repeats labeled. The inviable Tra1 deletions abolishing SAGA and NuA4 assembly are highlighted by the domain color scheme as in Fig. 2b. The viable Tra1 deletion region are indicated by gray pipes. **b,** Eight purple spheres on the Tra1 model indicate crosslinking sites to SAGA subunits determined by chemical crosslinking and mass spectrometry [10].

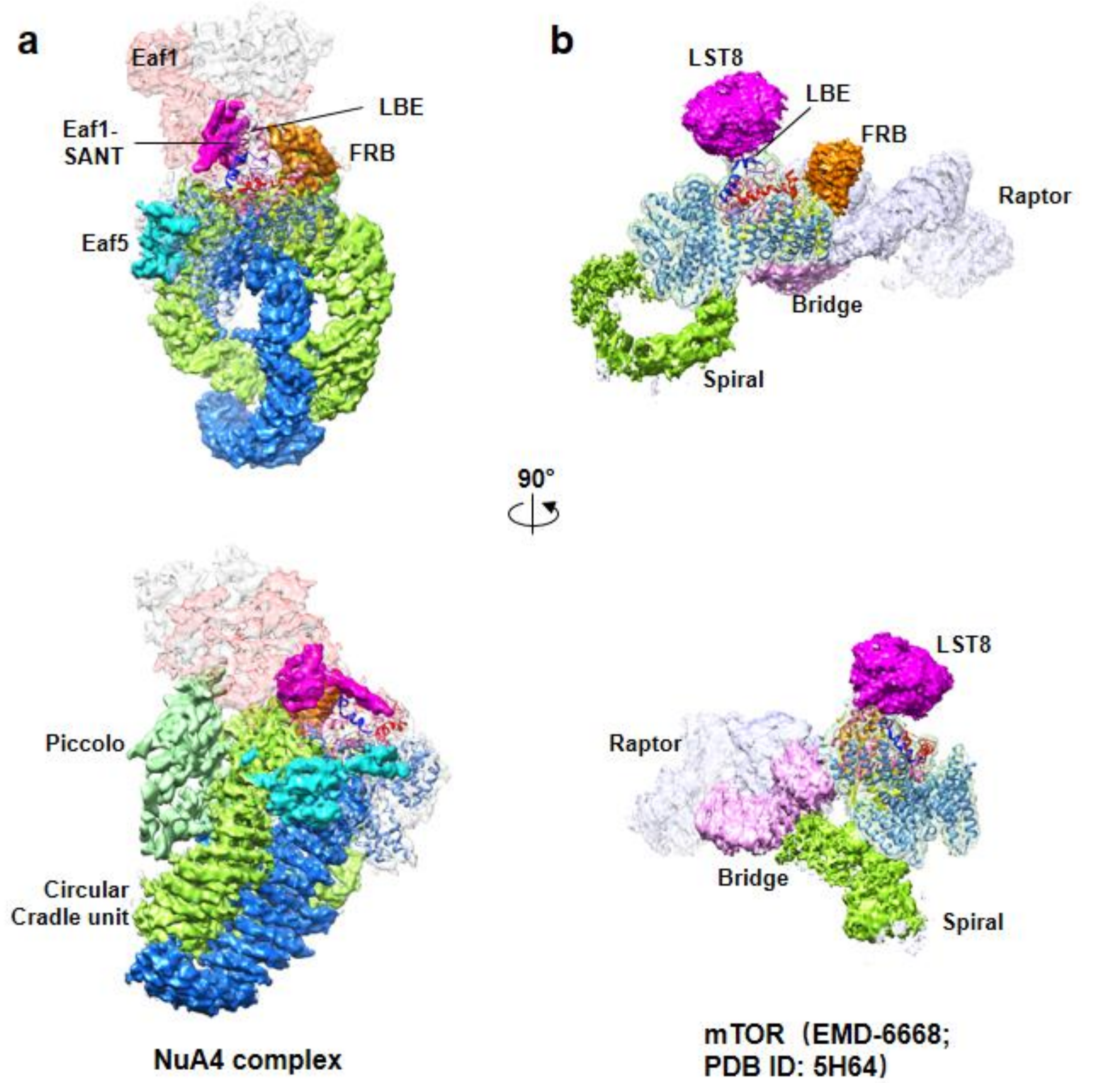

Supplementary Figure 16 | Comparison of Tra1 in NuA4 and mTOR in mTORC1. a, Two views of Cryo-EM structure of NuA4 fitted with Tra1 model. The color scheme of Tra1 is the same as in Fig. 2b. The cyan and magenta surfaces highlighted Eaf5 and the SANT domain of Eaf1. The SANT domain of Eaf1 directly interacts with LBE and FATC domains of Tra1. Piccolo colored in light green interacts with HEAT repeats of the Tra1. **b,** Cryo-EM structure of mTOR compelx 1 (EMD-6668) fitted with its model (PDB ID :5H64) [11,12]. The view of the two complexes are aligned according to the catalytic core of Tra1 and the mTOR. LST8 interacts with LBE domain. Raptor directly binds to Bridge domain of the HEAT repeats.

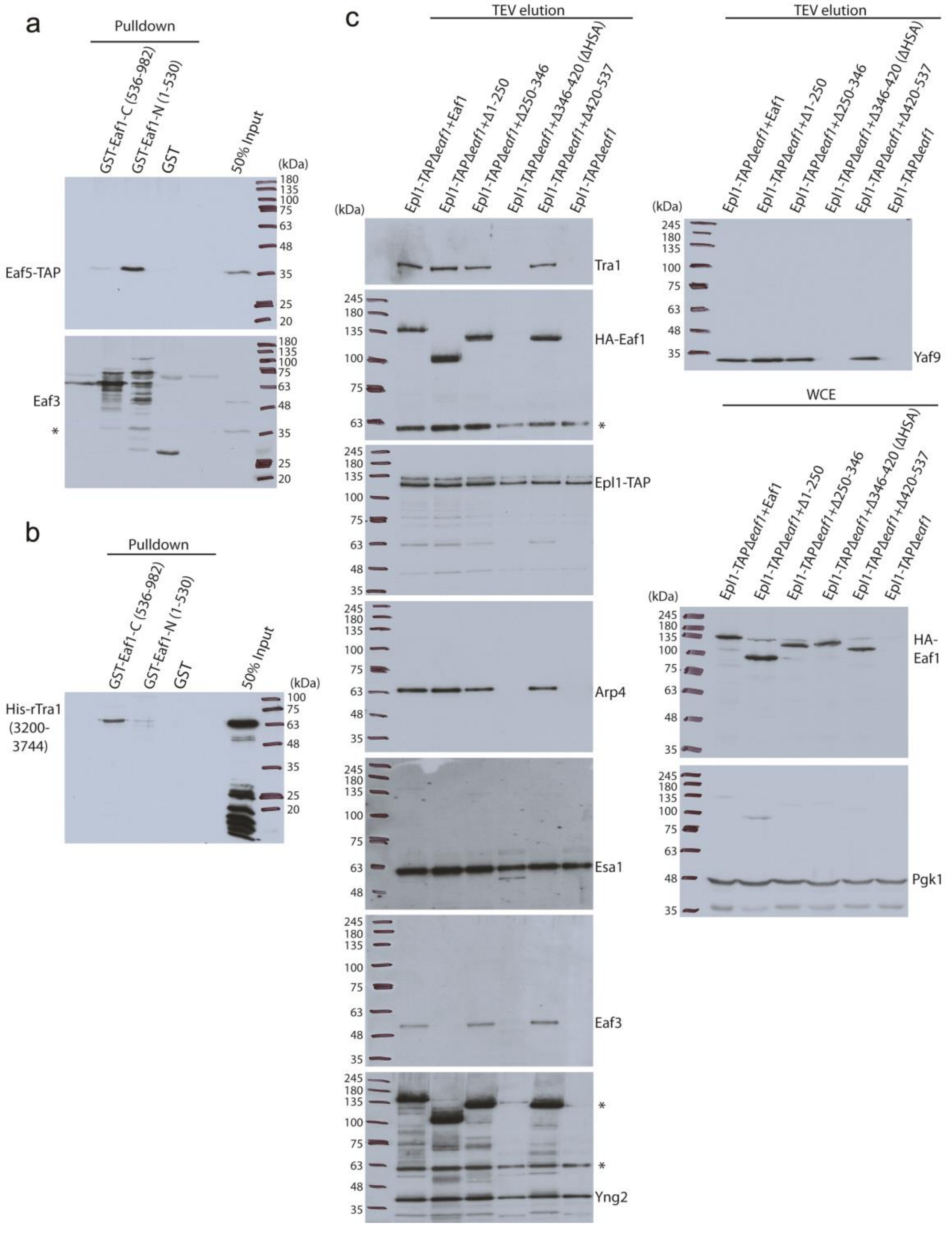

Supplemental Figure 17 | a, b, c Complete western blots corresponding to Fig 6a, 6b and 6c.

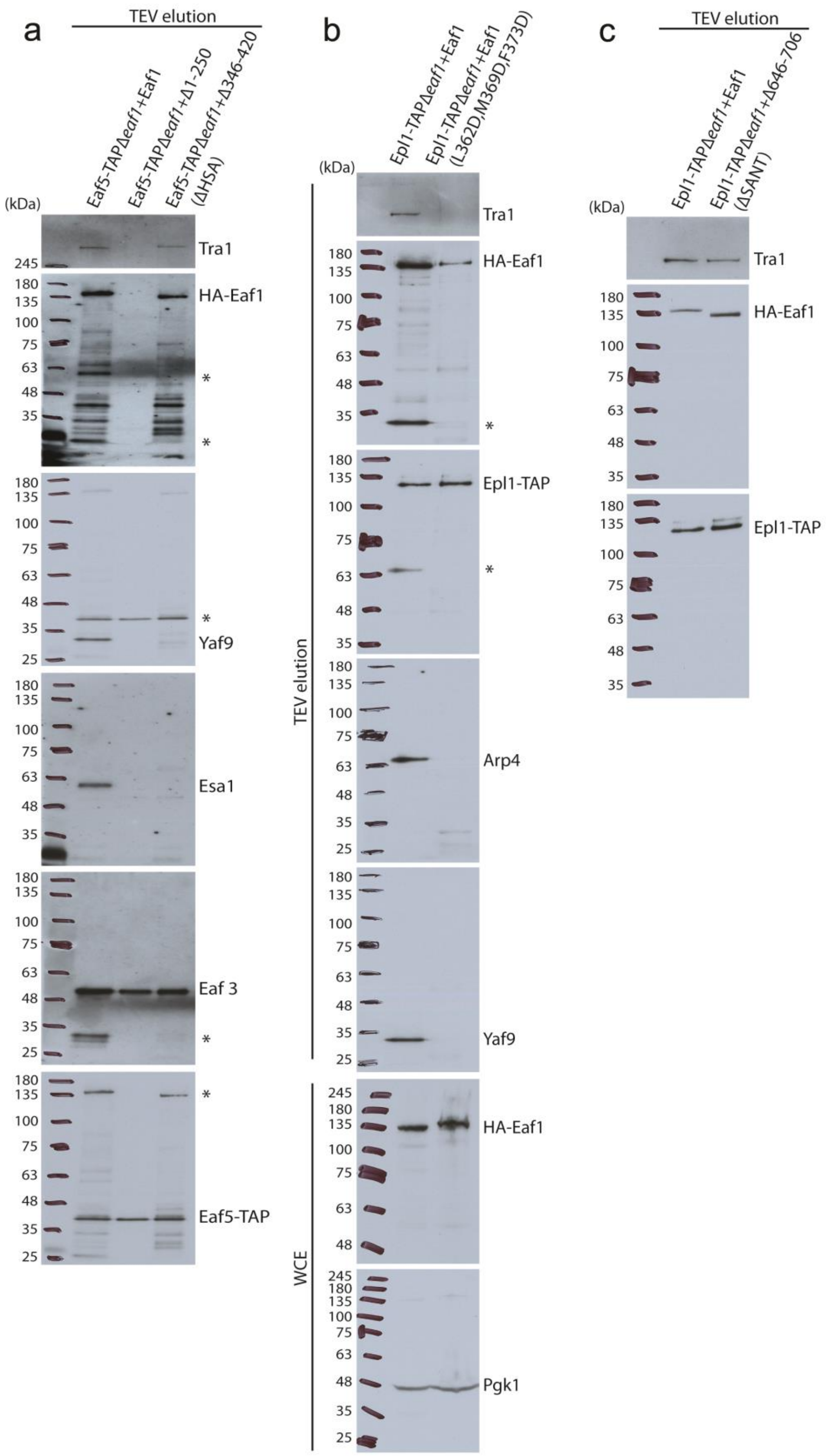

Supplemental Figure 18 | a, b, c Complete western blots corresponding to Fig 6d, 6f and 6g.

Supplementary Table 1. Statistics of 3D reconstruction and model refinement

	**TEEAA assembly (EMDB-6816, PDBID 5Y81)**	**TEEAA-piccolo assembly (EMDB-6815)**
**Data collection**		
EM equipment	FEI Titan Krios	FEI Titan Krios
Voltage (kV)	300	300
Detector	Gatan K2	Gatan K2
Pixel size (Å)	1.30	1.30
Electron dose ($e^-/Å^2$)	30	30
Defocus range (μm)	2.0~3.0	2.0~3.0
**Reconstruction**		
Software	RELION 2.0 (beta)	RELION 2.0 (beta)
Number of used Particles	63,197	19,060
Symmetry	C1	C1
Final Resolution (Å)	4.68	7.74
Map sharpening B-factor ($Å^2$)	-188.1	-344.5
**Model building**		
Software	Coot	–
**Refinement**		
Software	Phenix, refmac	
Average Fourier Shell correlation	0.9270	–
Rfactor	0.1958	–
**Validation**		
R.m.s deviations		
Bonds length (Å)	0.005	–
Bonds Angle (°)	0.86	–
Ramachandran plot statistics (%)		
Preferred	86.1	–
Allowed	8.16	–
Outlier	5.74	–

Supplementary Table 2. Mass spectrometry (MS/MS) analysis of TEV elutions from Epl1-TAP samples harboring various Eaf1 mutants, as shown in Fig. 6c. The values represent total spectral counts. Apparent loss of NuA4 subunits is shown in red and possible decreased interactions in yellow. Subunits are grouped into functional subcomplexes/modules. Other interactors shown include histones and Yap1 DNA-binding transcription activator [13].

		Eaf1						
	**NuA4 subunit**	**WT**	**Δ1-250**	**Δ 250-346**	**Δ 346-420 (HSA)**	**Δ 420-537**	**Δ Eaf1**	**R690A R691A E698A (SANT)**
	**Tra1**	388	354	315	2	396	0	254
	**HA-Eaf1**	161	148	146	4	176	0	145
**Piccolo NuA4**	**Epl1-TAP (bait)**	156	144	153	58	159	86	119
	**Esa1**	67	69	74	29	70	43	63
	**Yng2**	37	51	56	16	52	28	43
	**Eaf6**	5	7	8	2	8	4	5
**SWR1-C shared module**	**Swc4**	70	78	72	0	83	1	72
	**Arp4**	62	59	57	0	71	0	56

	**Yaf9**	25	33	28	0	34	0	29
**TINTIN**	**Eaf7**	18	0	14	0	26	0	17
	**Eaf3**	19	3	31	0	44	0	24
	**Eaf5**	20	0	23	0	35	0	20
	**Yap1**	5	8	1	0	7	0	0
	**H4**	5	2	4	5	4	5	4
	**H3**	1	2	2	1	2	2	2

Supplementary Table 3. Primer sequences used for cloning in the current study.

	Primer Sequence	Primer Target
1.	5’- ATAAGAATGCGGCCGCTCTAG -3’	F.P for Eaf1: Δ250-346aa, Δ346-420aa, Δ420-537aa, HSA- (L362D M369D F373D)
2.	5’- CTAGCTAGCTCTTTCTTTGA -3’	R.P for Eaf1: Δ250-346aa, Δ346-420aa, HSA- (L362D M369D F373D)
3.	5’- CTAGCTAGCTCGTCGTCAGAT -3’	R.P for Eaf1 Δ420-537aa
4.	5’- CTAGCTAGCCCCCCCATTTAAAC -3’	F.P for Eaf1: ΔSANT, SANT- (R690A R691A E698A)
5.	5’- GGGTATACTGTTTCTGGTTTGGT -3’	R.P for Eaf1: ΔSANT, SANT- (R690A R691A E698A)
6.	5’- GCTGCTCAGTGCGGCCGCGAGATAGATACATCAGACCATTATA -3’	F.P for Eaf1: Δ1-250aa
7.	5’- TAAATGGGGGGGCTAGCTC -3’	R.P for Eaf1: Δ1-250aa
8.	5’- TCCCCCCGGGGGTCCTCACGTCCAAGTTCA -3’	F.P for cloning Eaf1 1-530aa into pGEX-4T3
9.	5’- CCGCTCGAGTCAGATCAACGCTGATGATGCAG -3’	R.P for cloning Eaf1 1-530aa into pGEX-4T3
10.	5’- GAAGGATCCAAGAAAGAGCTAGCCCCC -3’	F.P for cloning Eaf1 536-982aa into pGEX-4T3
11.	5’- CTTCCTAGGACTAGTTTGTTTTTGGAACC -3’	R.P for cloning Eaf1 536-982aa into pGEX-4T3
12.	5’- TAGGAATTCCTCGAGCGGCGCCAGCCTTGGGAA -3’	F.P for cloning Tra1 3200-3744aa into pET15b
13.	5’- ACGACGCGGATCCCTAGAACCATGGCATGAAG -3’	R.P for cloning Tra1 3200-3744aa into pET15b

Supplementary References

1 Radermacher, M. Three-dimensional reconstruction of single particles from random and nonrandom tilt series. *Journal of electron microscopy technique* **9**, 359-394 (1988).

2 Pettersen, E. F. *et al.* UCSF Chimera--a visualization system for exploratory research and analysis. *Journal of computational chemistry* **25**, 1605-1612, doi:10.1002/jcc.20084 (2004).

3 Scheres, S. H. RELION: implementation of a Bayesian approach to cryo-EM structure determination. *Journal of structural biology* **180**, 519-530, doi:10.1016/j.jsb.2012.09.006 (2012).

4 Swint-Kruse, L. & Brown, C. S. Resmap: automated representation of macromolecular interfaces as two-dimensional networks. *Bioinformatics* **21**, 3327-3328, doi:10.1093/bioinformatics/bti511 (2005).

5 Diaz-Santin, L. M., Lukoyanova, N., Aciyan, E. & Cheung, A. C. Cryo-EM structure of the SAGA and NuA4 coactivator subunit Tra1 at 3.7 angstrom resolution. *Elife* **6**, doi:10.7554/eLife.28384 (2017).

6 Sibanda, B. L., Chirgadze, D. Y., Ascher, D. B. & Blundell, T. L. DNA-PKcs structure suggests an allosteric mechanism modulating DNA double-strand break repair. *Science* **355**, 520-524, doi:10.1126/science.aak9654 (2017).

7 Yang, H. *et al.* mTOR kinase structure, mechanism and regulation. *Nature* **497**, 217-223, doi:10.1038/nature12122 (2013).

8 Wang, X. *et al.* 3.9 A structure of the yeast Mec1-Ddc2 complex, a homolog of human ATR-ATRIP. *Science* **358**, 1206-1209, doi:10.1126/science.aan8414 (2017).

9 Knutson, B. A. & Hahn, S. Domains of Tra1 important for activator recruitment and transcription coactivator functions of SAGA and NuA4 complexes. *Molecular and cellular biology* **31**, 818-831, doi:10.1128/MCB.00687-10 (2011).

10 Han, Y., Luo, J., Ranish, J. & Hahn, S. Architecture of the Saccharomyces cerevisiae SAGA transcription coactivator complex. *EMBO J* **33**, 2534-2546, doi:10.15252/embj.201488638 (2014).

11 Aylett, C. H. *et al.* Architecture of human mTOR complex 1. *Science* **351**, 48-52, doi:10.1126/science.aaa3870 (2016).

12 Yang, H. *et al.* 4.4 A Resolution Cryo-EM structure of human mTOR Complex 1. *Protein Cell* **7**, 878-887, doi:10.1007/s13238-016-0346-6 (2016).

13 Mitchell, L. *et al.* mChIP-KAT-MS, a method to map protein interactions and acetylation sites for lysine acetyltransferases. *Proc Natl Acad Sci U S A* **110**, E1641-1650, doi:10.1073/pnas.1218515110 (2013).
